# Impact of COVID-19 on the neglected tropical diseases: a scoping review

**DOI:** 10.1186/s40249-024-01223-2

**Published:** 2024-07-29

**Authors:** Caitlin Brigid Butala, Roo Nicola Rose Cave, Jenna Fyfe, Paul Gerard Coleman, Guo-Jing Yang, Susan Christina Welburn

**Affiliations:** 1 Infection Medicine, Edinburgh Medical School: Biomedical Sciences, College of Medicine and Veterinary Medicine, The University of Edinburgh, 1 George Square, Edinburgh, EH8 9TB Scotland, UK; 2 Zhejiang University – University of Edinburgh Institute: Biomedicine, Zhejiang University School of Medicine, 718 East Haizhou Road, Haining, 314400 People’s Republic of China; 3 Key Laboratory of Tropical Translational Medicine of Ministry of Education, The School of Tropical Medicine, The First Affiliated Hospital, Hainan Medical University, Haikou, 571199 Hainan People’s Republic of China; 4 School of Global Health, Chinese Centre for Global Tropical Disease Research, Shanghai Jiao Tong University School of Medicine, Shanghai, 200025 People’s Republic of China

**Keywords:** Neglected tropical diseases, Funding, WHO, Research and development, Investment, Disability adjusted life years, COVID-19, Millennium development goals, Sustainable development goals

## Abstract

**Background:**

This study investigates the impact of the COVID-19 pandemic on the prevalence, management, and control of the neglected tropical diseases (NTDs) highlighting the current or prospective impact of COVID-19 on research and development funding for, and execution of, NTD programmes. This review was conducted to determine if, and how, NTDs were affected by COVID-19, and whether those effects will delay the elimination goals of the Sustainable Development goals.

**Methods:**

Using open-source available data from policy and documentation from official websites of the relevant stakeholders including but not limited to World Health Organization (WHO) documents and policies, government foreign aid documents, and the Policy Cures G-Finder reports, this scoping review explored ongoing challenges to supporting research and development (R&D) for the NTDs and in maintaining NTD control programs; examined the constraints posed for NTD management by the pandemic from disruptions to healthcare services, reduction of finance and explored the potential long-term implications and consequences for those poorer, neglected populations in low and middle income-countries (LMICs). This was done by a scoping review literature search, publications were subject to an initial practical screening step to ensure the most relevant publications were selected for full screening, with the focus on scoping the designated topic of the impact of COVID-19 on NTDs. We further undertook an evaluation of the socio-economic factors exacerbating the impact of COVID-19 on NTD burden.

**Results:**

Multiple disruptions and setbacks, likely to affect NTD programmes and progress towards their elimination targets were identified in this study. R&D funding for the NTDs and AIDs and TB has declined since the funding high point of 2019, and for malaria since the high point of 2018. Significant changes in allocation of R&D funding within the NTDs are observed post pandemic, likely because of prioritization among donors. Diseases for which the least R&D investment was reported in place, prior to the pandemic (mycetoma, taeniasis/cysticercosis, trachoma and Buruli ulcer) have been particularly impacted post pandemic. We identified specific NTDs including schistosomiasis, leprosy, and rabies that have been affected by the COVID-19 pandemic and disruptions caused to on ongoing NTD control and elimination programs. Pandemic restrictions disrupted essential medical supply manufacturing and distribution impacting immunization programs and hindered efforts to control the spread of infectious diseases. NTD programmes have experienced numerous setbacks including delays in mass drug administration programs (e.g. for schistosomiasis), cancelled or delayed vaccination programs (e.g. for rabies) and closure of testing facilities has resulted in reduced diagnosis, treatment, and disease elimination for all NTDs. Lockdowns and clinic closures causing disruption to essential healthcare services restricted NTD surveillance and treatment programs. Community fears around contracting COVID-19 exacerbated the constraints to service delivery. Disparities in global vaccine distribution have widened with LMICs facing limited access to vaccines and disruption to immunization programs. Finally, the pandemic has led to increased poverty with poor and marginalized communities, impacting nutrition, healthcare access and education all of which have long term implications for NTD management and control.

**Conclusions:**

The COVID-19 pandemic profoundly impacted global health research and global health equity. Attention and funding were diverted from all sectors, significantly affecting research and development efforts set out in the World Health Organization’s NTD elimination Roadmaps. Ongoing changes to funding, economic crises, logistics and supply chain disruptions as well as deepening poverty has put a strain on already weak healthcare systems and exacerbated LMIC healthcare challenges. In particular, the delays and constraints to NTD management and elimination programs will have long-reaching consequences highlighting the need for global cooperation and renewed investment to put the NTD roadmap back on track. Targets and milestones are unlikely to be met without significant investment for recovery, in place.

**Graphical Abstract:**

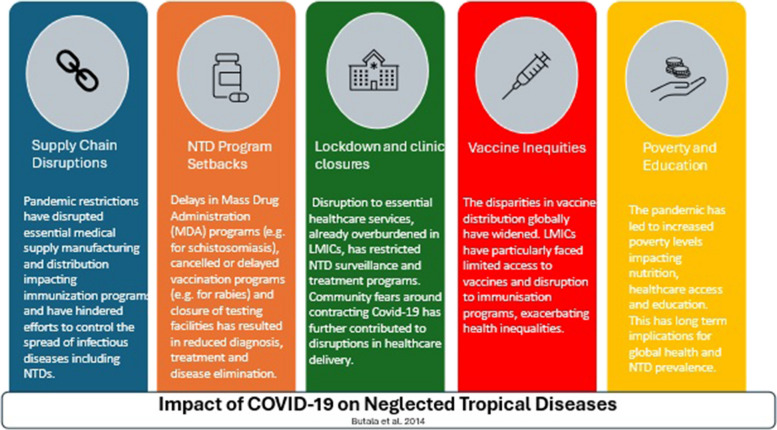

**Supplementary Information:**

The online version contains supplementary material available at 10.1186/s40249-024-01223-2.

## Background

Neglected tropical diseases (NTDs) affect poorer people in low-middle-income countries (LMIC). This NTD group has grown to include 21 types of disease which are considered neglected in comparison with tuberculosis, malaria and HIV/AIDS, also known as the Big 3 [1]. The Big 3 receive the bulk of funding in research and development dollars and the bulk of media coverage [2]. These 21 diseases can cause lifelong disabilities and impairments but historically garner less attention and funding than the Big 3; Tuberculosis, Malaria, and Human Immunodeficiency Virus/Acquired Immune Deficiency Syndrome (HIV/AIDS). The NTDs have lower mortality rates than the Big 3, but can lead to lifelong disfigurements, permanent changes to health, and the ability to work [3]. The reported lower global burden of NTDs is also reflects underreporting which is common amongst patients with NTDs due to stigma from the diseases or lack of reporting [4]. NTD programs are funded though governmental and non-governmental organizations (NGOs); these groups work together to fund intervention programs to stop the transmission of the diseases, as well as fund basic research and new treatments. Several high-income-countries (HIC) are at the forefront of foreign aid funding, usually pledging a commitment over several years. Global Aid funding to eliminate NTDs along with malaria, HIV, and tuberculosis aims to reach the World Health Organization (WHO) Roadmap 2020–2030 [5].

In 2019 the new coronavirus, SARS-CoV-2 made its debut in the world of global infectious diseases and took precedence in terms of funding, research, and public awareness. SARS-CoV-2 caused the disease now known as COVID-19, an infection in the upper respiratory tract that can cause serious illness and death [6]. COVID-19 became a global pandemic that has altered almost every aspect of daily life and at the time of writing (April 2024) has caused almost 7 million deaths worldwide, and 772.38 million cases [7]. COVID-19 has understandably been prioritized, this was particularly crucial in the early days of the pandemic as scientists, public health experts and governments tried to understand the new disease. This focus also impacted the research and development world, eating into funds that were previously designated for NTDs, the big three, and other common infectious diseases.

Research into treatments for COVID-19, the development of vaccines and research into the repurposing of existing drugs to treat severe COVID-19 have been very successful, but the cost has been high [8–10]. Academic research grants related to COVID-19 were abundant with the total amount exceeding USD 2.6 million [7, 11]. Governments and private donors contributed more money to these grants accounting for an increased portion of funding. The Global Health report states that in total an estimated USD 243.8 billion has been committed to COVID-19, although only USD 139.1 billion has been disbursed and of that only USD 13.7 billion has been for health-related work [12]. COVID-19 has had a crippling effect on NTDs from several fronts and angles. Like many aspects of eliminating NTDs, this is a multi-dimensional problem that requires a complex solution. This aims to inform a commentary on the impact COVID-19 has had on the research and development efforts set out in the WHO’s NTD elimination Roadmaps.

## Methods

This study analyzed and evaluated a wide range of sources to obtain evidence-based information to explore the challenges posed by the pandemic in maintaining existing NTD control programs, the disruptions to healthcare services, reduction of R&D finance and the potential long-term implications and consequences for those poorer, neglected populations in LMICs disproportionately affected by the NTDs. We aimed to identify specific NTDs affected by the COVID-19 pandemic, and disruptions caused to ongoing NTD control and elimination programs. We further undertook an evaluation of the socio-economic factors exacerbating the impact of COVID-19 on NTD burden, using open-source available data.

This scoping review was conducted in full accordance with the JBI methodology for scoping reviews. Search strategies included database searches, hand searches and application of snowball methodologies as outlined below following specific inclusion and exclusion criteria as outlined below. Full details of the research methodology can be found in Additional file 1.

### Search strategy

#### Database searches

Searches were run in the following databases: PubMed, Web of Science, JSTOR, Science Direct, and Google Scholar.

The searches were constructed by combining search terms from Additional file 2. For the NTD and Big 3 searches respectively one or more search term was used from each word group. Words within a word group were combined with OR, AND, and, were used between word groups. The initial searches for the 20 NTDs included by WHO between 2000‒2003 were performed in November and December of 2020 with follow up searches in June 2021, September 2022, and December 2023. An additional search was undertaken in April 2024 following the addition of noma, officially included as the 21st NTDs in late December 2023, but no additional sources were identified.

Publications were subject to an initial practical screening step to ensure the most relevant publications were selected for full screening, with the focus on scoping the designated topic of the impact of COVID-19 on NTDs. Practical screening assessed the topic of the publication as well as date of publication.

**Fig. 1:**
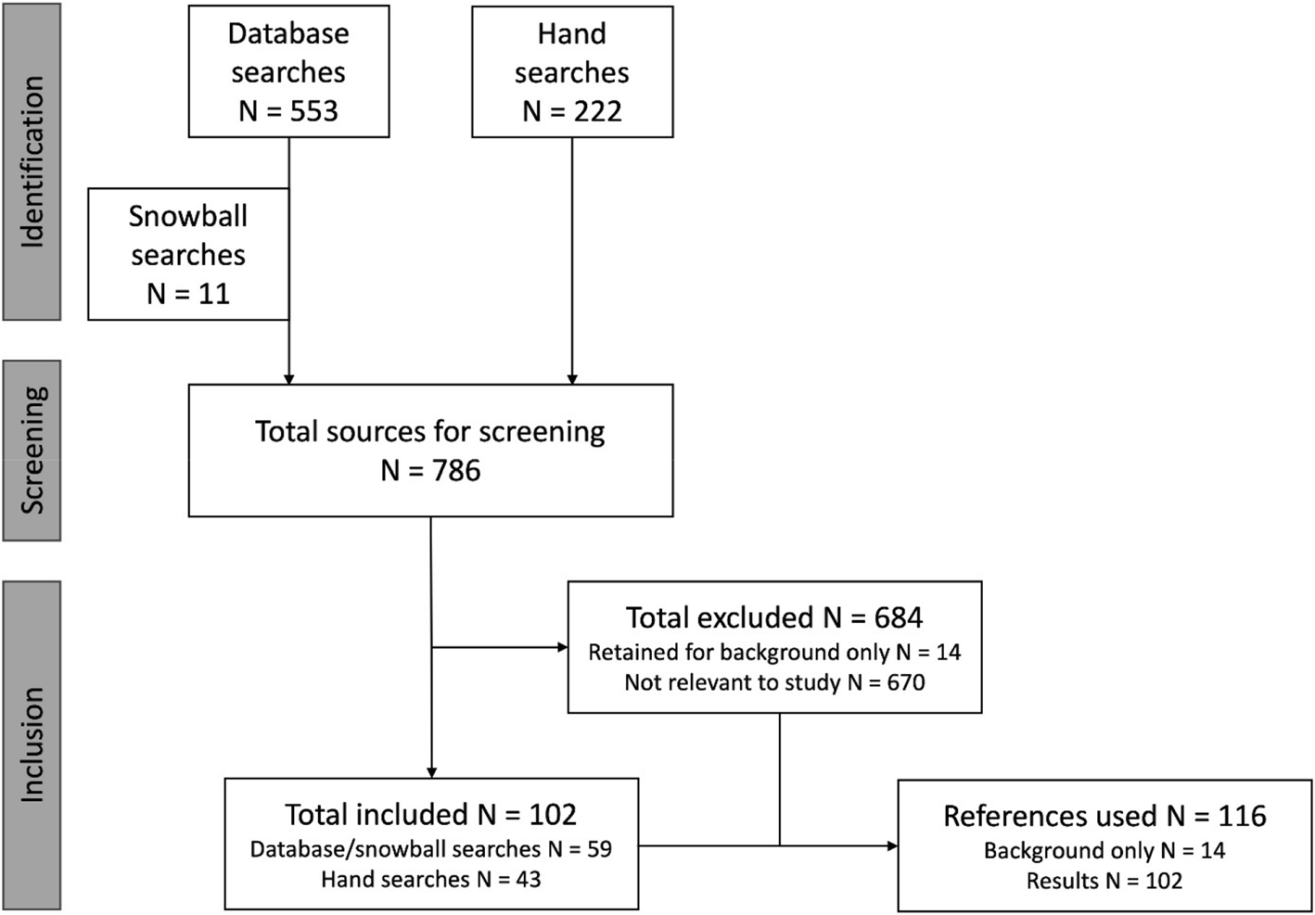
Flow diagram of search profile for scoping review

In total 553 publications were extracted for title and abstract and full text screening (see Additional file 2). Figure 1 shows a flow diagram of the inclusion and exclusion process.

#### Hand searches

The literature/data included in this study were found using open-source available data from policy and documentation. Official websites of the relevant stakeholders such as NGOs, LMIC governments, philanthropic groups and countries giving foreign aid were searched for news or policy updates to their NTD work.

Information was also obtained by searching through government foreign aid policies, grant proposal and awards, NGO annual report statements, and often news articles obtained through Google Search. While news articles are not typically used in journals or academic work, this information is not available from academic sources yet, and wherever possible academic articles were used instead of news articles. When selecting these sources, key words were used, including any projects mentioning water, sanitation, and hygiene (WASH), NTD, Neglected Tropical Diseases, or any specific disease name on the WHO NTD list in relation to COVID-19 or budget cuts.

In total 222 information sources were compiled through this method to be screened.

#### Snowball method

A subsequent search of the bibliographies of the articles selected for full review was also conducted using a snowball method, using the same inclusion and exclusion criteria applied to the original search. The search and subsequent analysis were carried out by the primary author of this review. 11 articles and sources were found through this method for screening as seen in Fig. 1.

### Inclusion and exclusion criteria

From the database, snowball and hand searches 786 publications were extracted for title and abstract and full text screening.

Inclusion criteria was health policy makers, health programs, ministries of health, NGOs, philanthropists, Official Development Aid donor countries that are working in connection with NTDs; health policies, programmes, interventions, diagnostics, treatments, and management focused on NTDs; work contributing to the management, monitoring, or elimination of NTDs where the impact of COVID-19, negative, positive, or neutral must be discussed.

Exclusion criteria were papers including health policies, health programmes, interventions, diagnostics, treatments, management not addressing diseases listed as NTDs where there was discussion of work on NTDs but with no information or discussion of finance and the impact of funding on NTDs and/or COVID-19.

### Analysis of yearly research and development funding data (USD) for NTDs

**Fig. 2:**
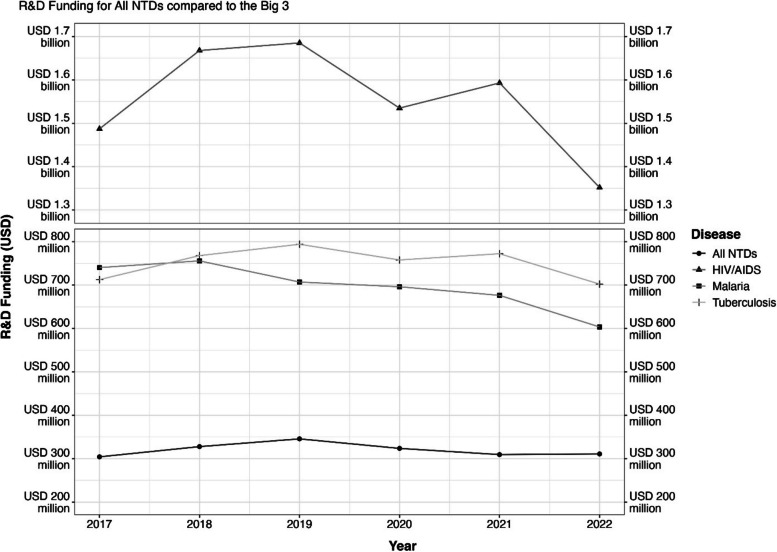
R&D funding for all NTDs compared to the Big 3 from 2017 to 2022. Note: Tracking of NTD Research and Development funding in comparison to HIV/AIDS, malaria and tuberculosis funding for the years 2017‒2022. R&D funding data for all NTDs from 2007–2022 can be found in Additional file 2

**Fig. 3:**
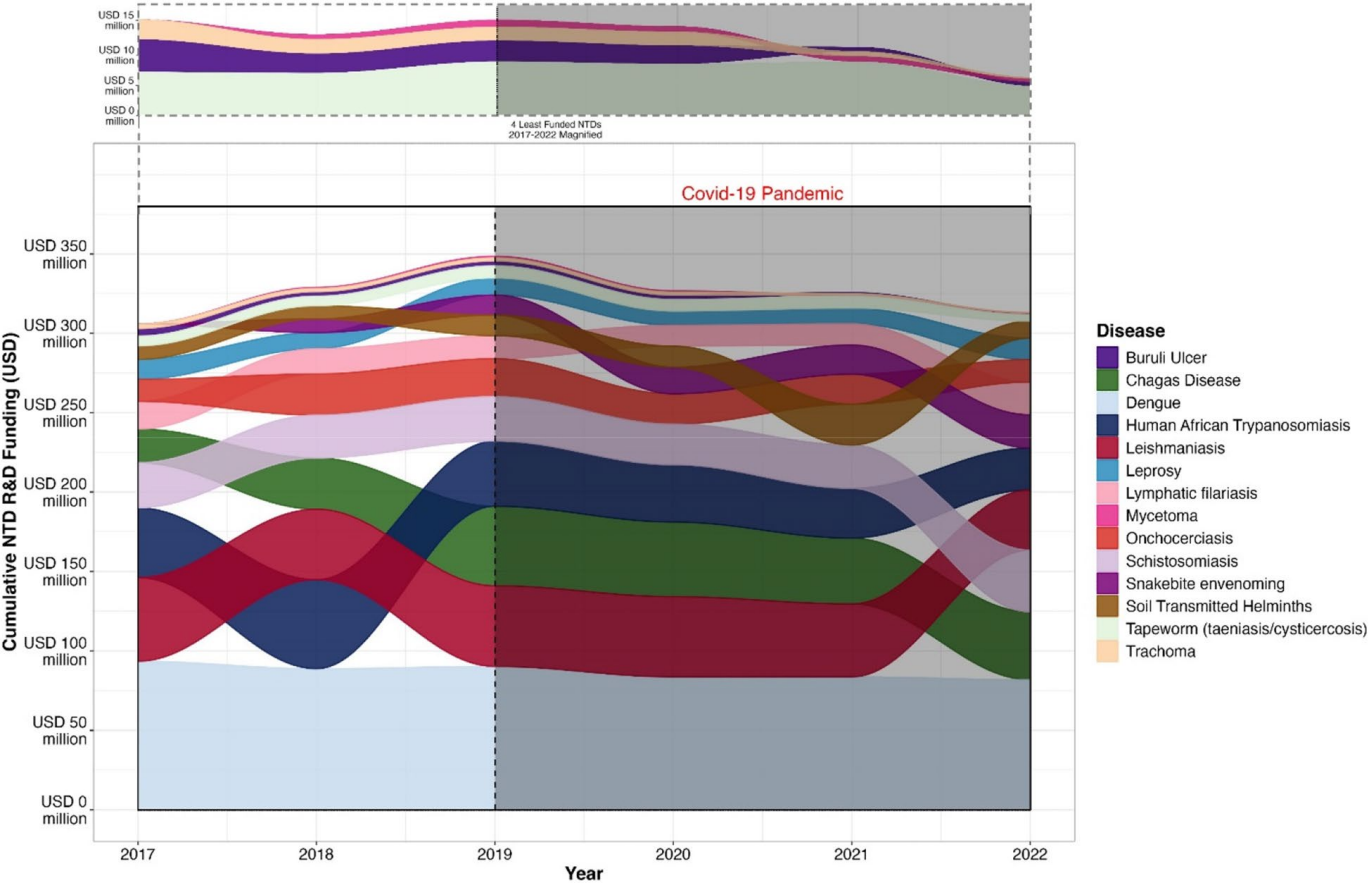
Alluvial plot illustrating the research and development funding spent (USD) for 14 neglected tropical diseases (NTDs) between 2017 and 2022. The colours represent the NTDs and the alluviums are arranged in decreasing order of proportion of the overall of funding demonstrated by the width of the alluviums. The diseases represented are as follows: mycetoma, taeniasis/cysticercosis, trachoma, Buruli ulcer, leprosy, lymphatic filariasis, Chagas, soil transmitted helminths, schistosomiasis, human African trypanosomiasis, dengue, leishmaniasis. The crossing of alluviums over time shows the changing prominence of allocated funding for each disease. The COVID-19 Pandemic is highlighted. The 4 least funded NTDs of the 14 for which data is available (mycetoma, taeniasis/cysticercosis, trachoma, Buruli ulcer) are magnified in the top panel to allow greater clarity. Note: R&D funding data for all NTDs 2007–2022 can be found in Additional file 2

To explore changes in funding profiles over time, pre and post COVID-19, for the NTDs, a quantitative analysis of yearly research and development (R&D) funding data, in US dollars, was undertaken in which data was extracted from the Policy Cures G-Finder Report on January 31, 2024, for the following diseases: mycetoma, taeniasis, cysticercosis, trachoma, Buruli ulcer, leprosy, lymphatic filariasis, Chagas, soil transmitted helminths, schistosomiasis, human African trypanosomiasis (HAT), dengue, leishmaniasis. The funding for taeniasis and cysticercosis was combined due to the data being combined in the G-Finder data portal. The soil transmitted helminth funding was the combined funding for whipworm (trichuriasis), roundworm (ascariasis), hookworm (anclyostomiasis and necatoriasis) and strongyloidiasis. All data was then graphically represented as a line plot or alluvial plot produced in in R (version: R 4.3.1 GUI 1.79 Big Sur ARM build, https://cran.r-project.org/) with R Studio (version: 2023.06.1 + 524, 2023.06 Mountain Hydrangea, Built on July 6, 2023 from 547dcf86, https://github.com/rstudio/rstudio/commits/547dcf861cac0253a8abb52c135e44e02ba407a1) using) using ggplot2 and ggalluvial packages shown in Figs. 2 and 3.

## Results

Using open-source available data from policy and documentation from official websites of the relevant stakeholders including but not limited to World Health Organization (WHO)_ documents and policies, government foreign aid documents, and the Policy Cures G-Finder reports, this scoping review explored ongoing challenges to supporting research and development (R&D) for the NTDs and in maintaining NTD control programs; examined the constraints posed for NTD management by the pandemic from disruptions to healthcare services, reduction of finance and explored the potential long-term implications and consequences for those poorer, neglected populations in LMICs and evaluated the socio-economic factors exacerbating the impact of COVID-19 on NTD burden.

Of the 786 publications, 102 were included for the scoping review analysis. Of the 684 excluded, 14 were retained for background only.

### The impact of COVID-19 on NTDs

In 2019 the new coronavirus, SARS-CoV-2 knocked even the Big 3 to a lower status. SARS-CoV-2 is a novel coronavirus that causes COVID-19, an infection in the upper respiratory tract that can cause serious illness and death. COVID-19 is a global pandemic that has altered almost every aspect of daily life and at the time of writing has caused more than 6.88 million deaths worldwide [11]. COVID-19 has understandably been prioritized and taken over the research and development world as well, eating into funds that were previously designated for NTDs. Research into treatments for COVID-19, the development of vaccines and the repurposing of existing drugs has been very successful, but the cost has been high. Academic research grants related to COVID-19 were abundant with the total amount exceeding USD 2.6 million [11]. Governments and private donors contributed more money to these grants accounting for an increased portion of funding. The Global Health report states that in total an estimated USD 243.8 billion has been committed to COVID-19, although only USD 139.1 billion has been disbursed and of that only USD 13.7 billion has been for health-related work [9, 13]. Furthermore, the USD 786.6 million contributed to development assistance for pandemic preparedness in 2021 was a 64.8% increase from the 2019 contribution. More than 97% of this 2021 funding lacked the geographical detail to be disaggregated to global, regional, or national services. Pandemic preparedness development assistance funding peaked in 2020 at USD 1049.6 million, future results will determine if funding continues to decrease [9, 14–18].

### Funding for R&D for NTDs rerouted to COVID-19 research

Overall, funding for COVID-19 and future pandemic preparedness has dwarfed R&D funding for the NTDs. In terms of R&D funding, an estimated 98.12% of the total USD 5.9 billion initially allocated to COVID-19 research has largely been publicly funded by central governments [14]. Of USD 9.18 billion pledged or funded, the bulk of this money has been for vaccine developments (USD 5.5 billion), therapeutic treatments research (USD 1.3 billion), diagnostics (USD 804 million), basic research (USD 212 million), with USD 1.3 billion for unspecified categories [15]. Bill and Melinda Gates Foundation has donated USD 1.75 billion to COVID-19; most allocated to development of vaccines, diagnostics, and treatment drugs [16]. The Wellcome Trust, in the first year of COVID-19 awarded 28 grants totaling GBP 24 million [17]. World Report, shows that for all coronaviruses considered and/or COVID-19, a USD 183 million spend since 2020 [18].

The largest private single source of funding for COVID-19 is from the Bill and Melinda Gates Foundation, who are the biggest single private funder for NTD research. The Foundation has clearly stated that funds spent on COVID-19 research has not been diverted from other pledged projects, and are the only funder to have made an open statement about their funding intentions [19]. R&D funding for the NTDs and AIDs and TB has declined since the funding high point of 2019, and for Malaria since the high point of 2018 (see Fig. 2).

Notably, R&D funding for NTDs after the London Declaration in 2012, a pledge from governments, industry, and philanthropists to commit to “*control, eliminate or eradicate 10 diseases by 2020 and improve the lives of over a billion people*” did not improve as anticipated post declaration, and in the case of several NTDs R&D funding decreased after 2012 (see Additional information file 2). This is representative of the R&D funding, not intervention, or control programming and funding may have been targeted at control rather than R&D although arguably for the NTDs R&D and control are intwined. For the years of 2012–2017 R&D funding is observed to rise and fall but post 2017 there is consistent rise in funding leading to record funding level in 2018 and 2019, however, as observed in Fig. 2, funding levels fell again thereafter.

Data shows that funding for R&D for the NTDs is varied, changing year to year as a grouping and as individual diseases. As shown in the alluvial plot (Fig. 3), funding for R&D for the NTDs has in the main remained relatively stable, but the proportions of the overall funding allocated to an individual disease can be observed to shift from year to year, especially in pre- and post- pandemic periods.

Out of the 15 NTDS that the G-Finder lists within its data portal, 3 diseases have been newly added to the NTDs. Snakebite envenoming is an outlier due to its inclusion into the NTD group in 2017, which shows an increase over the 5 years based on the inclusion and increased awareness that may follow inclusion. Scabies and Mycetoma showed similar albeit smaller increases since their inclusion to the NTD group in 2017. Nine diseases (dengue, Chagas, leishmaniasis, human African trypanosomiasis, onchocerciasis, taeniasis, Buruli ulcer, trachoma and soil transmitted helminths) all showed decreased R&D funding post COVID-19 pandemic as would be expected. Three diseases showed small increases in R & D funding between 2020 and 2021: schistosomiasis (up by 6.5%), lymphatic filariasis (up 1.55%) and leprosy (up 10.4%). Larger increases in funding were observed between 2021 and 2022 for schistosomiasis (increased by 29%), lymphatic filariasis (increased by 35%) and leprosy (increased by 27%). R&D funding for schistosomiasis, leprosy, and lymphatic filariasis has rebounded since 2020 reaching their peaks levels for the previous 5 years in 2022. Chagas R&D funding increased in 2022 but not to pre-2020 level funding. Funding for the remaining 8 NTDS has continued to decrease in the post-COVID-19 era.

Despite record funding for NTDs, every year the funding gap between the amount donated and the amount needed to meet and maintain target goals towards elimination within the roadmap widens [20]. The costs needed to reach elimination targets increase as the numbers of people affected decrease. This is due to having to travel to reach more rural areas, maintain a longer cold chain to transport vaccines and medicines and the fact that cost/benefit goes down as you decrease the number of people being treated. The converse of this scenario can also lead to an increase in costs, these higher costs are connected to increasing populations at risk due to an increase in poverty, increase in vector habitat due to climate change, and an increase in populations [21–24].

### UK foreign AID budget cuts

Governmental funding of NTD research and control programs is vital to ending transmission and the eventual elimination of NTDs. HICs donate money through their foreign aid budgets to fund NTD programs, and LMIC fund programs through their governmental budgets. Until recently, the USA and the UK have been the biggest governmental donors to foreign aid budgets.

**Fig. 4:**
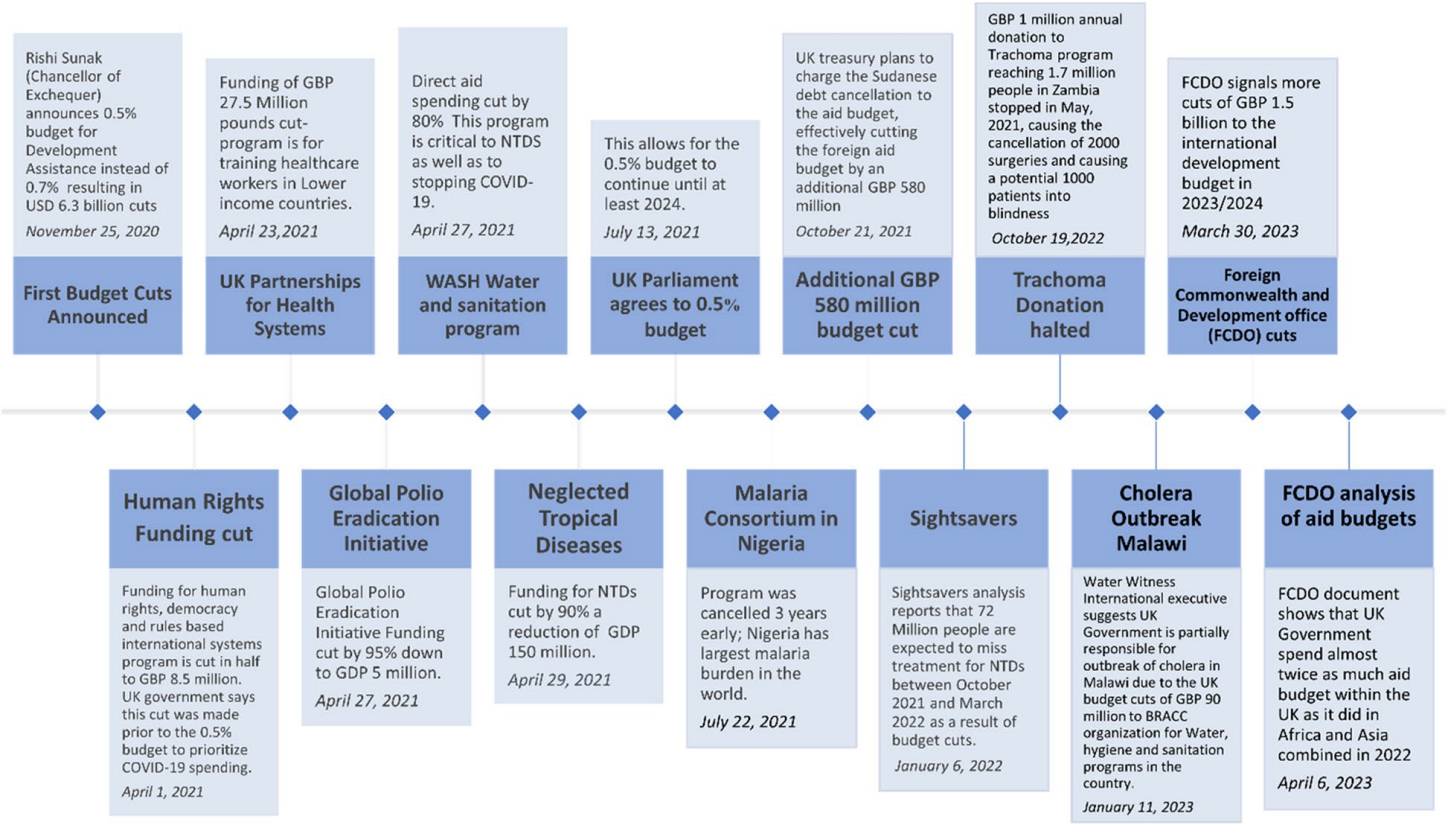
Timeline of UK foreign aid budget cuts

In November 2020, after declaring that the United Kingdom, through UKAID, would continue to fund NTDs, the UK announced that the UKAID budget would be cut dramatically from 0.7% of the gross national income to 0.5% [25]. There has been speculation as to whether the budget cuts were due to ‘Brexit’ or the pandemic, however, the reason given by the government for the cuts was the “economic hurricane” of the pandemic and the amount of money that the UK government had spent on the pandemic [26]. Figure 4 shows the timeline of announced budget cuts and the effects on NGO programs. This budget cut meant that many NGOs that were already recovering from time away from their programs due to travel restrictions and lockdowns installed in response to COVID-19, now also face financial shortages that will affect their ability to supply aid in NTD endemic countries. NGOs contribute money, labor, and organizational infrastructure in areas they work in to improve the health of patients and affected individuals in those communities, without NGO interventions, much of the work of eliminating NTDs would be expected to fall further behind.

A key part of core funding for NTDs is the provision of infrastructure for water and sanitation. Water, sanitation and hygiene (WASH) is important as access to clean water and appropriate sanitation is vital for the elimination of several NTDs [27, 28]. Different aid groups and diseases have been affected by the UK cuts, with clean water, sanitation and hygiene budget being cut by 80% [29]. The executive director of the Water Witness International group came forward to suggest that the UK should be held responsible for the cholera outbreak in Malawi which has killed 1210 people after the UK cut the WASH aid budget of the country by GBP 90 million [30]. The UK has since contributed GBP 500,000 to the Malawi relief fund, but the total funding needed is GBP 14 million [30]. While the UK is not the only country that donates money through aid programs, it was the 2nd largest contributor, with this cut dropping it 4th, below France and Germany in the ranking of donors. Other countries and pharmaceutical donors may decide to stop funding aid or donating medicine, knowing that the medicines will expire before being distributed. The Trump administration in the USA proposed several cuts to foreign aid since 2016, ranging from a 20‒30% decrease in foreign spending. In no year during that period did the proposed budget cuts pass, however, it may have contributed to the UK government’s willingness to reduce their aid budget.

### Filling the void

Considering the UK foreign aid budget cuts, several independent philanthropic donors have stepped in to fill the void. The Bill and Melinda Gates Foundation, the Children’s Investment Fund Foundation, the ELMA Foundation, and the Open Society Foundations have pledged GBP 94 million to cover part of, but not all the cuts to the budget. This amount will help to cover the immediate costs to keep some clinics open and drug disbursement until the budget is restored or other funding can be found by NGOs [31]. The chief executive of the Children’s Investment Fund Foundation said: “These life-saving treatments are cost-effective investments. If they go unfunded this year, British taxpayer generosity will be wasted as clinics are closed and essential drugs expire and are thrown away” [31]. Despite the GBP 94 million supplement from private donors, some programs will still have to close due to lack of funding. One such programme, Accelerating the Sustainable Control and Elimination of Neglected Tropical Diseases (ASCEND), will close their doors and cancel lifesaving programs. ASCEND has been working on visceral leishmaniasis (VL) in Ethiopia, Kenya, Sudan, South Sudan, Uganda, Nepal and Bangladesh, and the closing of that program is projected to result in an additional 20,000‒30,000 deaths from VL [32].

### COVID-19 impacts on supply chains

COVID-19 restrictions alongside budget cuts resulted in donated medicines sitting in warehouses in LMIC and expiring because the NGO workers could not enter the countries or could not pay to have workers already in the country distribute those medicines [33]. Distribution requires transportation, translators, cold chain storage for some vaccines. Mass drug administration (MDA) programs, which often rely on schools to administer the drugs to children, have already been delayed a year in many cases due to school closures, and now risk further delays. The NTD Modelling Consortium presented models that a 12-month delay of MDA will set the elimination of the disease back another three years. Due to these budget cuts, many programs are facing two to three years setback, causing a 6-to-9-year elimination delay. These MDA programs have subsequently resumed in some NTD endemic countries. However, these programs face continue to face challenges due to ongoing resource shortages. It is also reported that post COVID-19 pandemic some communities are reticent to engage with health care services due to transmission fears [34, 35].

COVID-19 has also had a considerable effect on supply chains and manufacturing of drugs, through lockdowns preventing the manufacturing and shipping of produced goods. Vials used for vaccines are made from borosilicate glass and need to be made to certain specifications, but the current vial making glass companies were focused on providing enough vials to contain vaccines [36]. At two doses per person, that is fifteen billion vials, for COVID-19 vaccines alone, not including those required for “routine” vaccines globally. This represents the bulk of the global production of vials which runs between 15 and 20 billion [36]. This shortage has also affected blood testing in the UK, in August 2021, the NHS had to suspend some blood testing due to lack of testing vials [37].

In October 2021, WHO reported there was a more than 2 billion shortfall on syringes, specifically the 0.5 ml auto-disable syringes used for COVID-19 vaccines and routine immunization vaccines and the 0.3 ml auto-disable syringes used for the Pfizer BioNtech COVID-19 vaccine. This shortage will not only impact the ability of LICs to vaccinate for COVID-19 but other lifesaving vaccinations like measles, malaria, and polio (which all require the 0.5 ml syringe); while rabies post exposure requires a larger 1.0 ml syringe, although these can be given without the auto-disable syringe, WHO recommends single use to prevent cross contamination and infections [38–40]. This has become even more urgent as wild poliovirus has been reported in Malawi for the first time since 2016, and vaccination will be essential to prevent further spread of polio [41].

COVID-19 also affected supplies of personal protection equipment which prevent the spread of disease, as well as hand sanitizers and soaps, both of which are crucial to stopping the spread of half of all NTDS. For a large part of 2020 there were not enough face masks being produced leading people to make their own masks at home out of cloth or face shields out of recycled plastic bottles. These masks were less effective than medical supply masks or K95 masks leading to greater risk of infection [42]. The Federal Drug Administration (FDA) of the United States maintains a medical supply shortage list that continues to have basic medical necessities like surgical gloves and gowns listed as in short supply [43].

### Consequences of the COVID-19 pandemic to progress for TB and malaria

TB setbacks and similar will cost in the long run to catch up to where we were. Tuberculosis (TB), one of the Big 3, has seen quantifiable setbacks to treatment and elimination projects around the globe [44, 45]. This can be used as a canary in a coal mine to determine funding health for more neglected NTDs, if the Big 3 are losing funding and being setback by the pandemic we can extrapolate the effects on the remaining NTDs. TB is closely linked to NTDs through the Millennium Development Goals (MDGs) and the Sustainable Development Goals (SDGs) [46, 47].

TB receives more funding both in research and development dollars and in donations from NGOs and foreign aid than all the NTDS, save malaria and HIV/AIDS. On any given year since the GFinder reports began in 2007, TB has received more than double the research and development dollars than the next closest non- big 3 NTD (diarrheal diseases) [48, 49]. Human African trypanosomiasis, leishmaniasis, and other diseases receive so little funding as to be labeled “unspecified diseases” on the GFinder reports [48].

Limited studies available on co-infections of TB and COVID-19 are often contradictory, with one study suggesting that a latent TB infection boosts the immune system and helps lessen the severity of COVID-19 infections, with another suggesting that the effects of COVID-19 more than double a person’s chances of dying from COVID-19 [50, 51]. There are no contraindications on treating TB and COVID-19 at the same time. TB management NGOs have recommended sending TB patients home with enough medicine to continue to treat at home removing the need to risk contracting COVID-19 while seeking treatment for TB [52, 53].

COVID-19 has affected both active case finding and treatment of already diagnosed cases. This is represented by the numbers in India, National TB Programmes (NTP) report an approximately 80% decline in the daily TB notifications, reporting new cases to health authorities [54]. A survey conducted by the Global Coalition of TB Activists shows that 40% of NTP are being used as COVID-19 response centers [55]. This creates a lack of space, workers, time, and lab space to diagnose new cases of TB, or to treat those already diagnosed cases.

This contributed to people not seeking treatment for their ongoing TB infections, fearing COVID-19 infections in the hospitals and clinics that usually serve TB patients but are now also treating COVID-19 patients [56]. Although the connections are still being studied, the comorbidities of TB and COVID-19 and the severity of subsequent infections present a unique hazard. Since both diseases cause similar symptoms of difficulty breathing and coughing, the scarification of lungs of TB patients stands to be further damaged by COVID-19, as well as making patients more susceptible to infection [57].

TB clinics had to close due to lockdowns in several endemic countries, this delayed treatment of people willing and able to receive treatment, contributing to more transmission, reactivation of TB in patients, longer time of treatment and further TB complications [58, 59]. Stopping treatment, whether due to inability to obtain medicine or by choice, also increases the likelihood of drug resistance, a growing problem even with rigorous treatments. TB is fatal if left untreated, and even in the pre COVID-19 era roughly 4000 people died per day of TB [53].

TB is not an outlier in the NTD community, if one of the most well-known, and well-funded “other diseases” featured in the MDGs is being affected financially and with disruptions of treatment, it signals worse indications for the neglected of the neglected diseases. Global cessation of TB transmission has been a goal since the beginning of the MDGs, which has carried through to the SDGs [60]. COVID-19 has presented new unforeseen challenges to this goal and has set progress back by several years due to interruptions of treatment, active case finding and diagnostics. According to the Global fund annual report for 2021, TB cases were down 18% which on the surface seems encouraging but the reason for the decrease is less testing and less active case finding meaning that more people are living with the disease instead of receiving treatment [61]. The percentage of countries reporting disruptions to TB diagnosis and treatment increased from 40% (of 124 countries) in 2020 to 51% (of 98 countries) in 2021 [62].

As is the case for TB, malaria as one of the Big three receives more attention and funding than any neglected tropical disease by at least two-fold in any given year. Even with this advantage, malaria has faced setbacks in terms of progress towards elimination and reducing cases and deaths. In 2019 there were an estimated 227 million cases of malaria worldwide, already up from the 2015 low of 224 million. In 2020, that number has increased to 241 million cases, predominately attributed to the service disruptions caused by COVID-19 [63]. These disruptions include control programs to distribute insecticide treated bed nets, indoor residual spraying, and seasonal malaria chemotherapy campaigns. in 2020 there were 37 of 64 (58%) responding endemic countries reporting disruptions to diagnosis and treatment, this dropped to 23 of 59 responding countries in 2021. Insecticide treated net distribution disruption is still at 19 endemic countries reporting disruptions, 14 countries reporting disruption of indoor residual spraying [62]

The number of countries reporting disruptions has decreased between 2020 and 2021, however there are still many endemic countries reporting disruptions at various severity levels, and all disruptions affect the ability to reach the Global Technical Strategy Target for 2030 [64].

### Consequences of COVID-19 for control and elimination of the NTDs

In the early stages of the pandemic between March and August of 2020, several models were developed to predict the outcome of delays and cancellations of NTD programs over time. Schistosomiasis MDA programs were modelled showing that the delay towards elimination as a public health problem would only be delayed for the same amount of time as the delay of the distribution of medicine. This does not account for areas with higher transmission or programs that are in later stages of running where the risk is losing the long-term benefit of multiple rounds of MDA to stop transmission [65, 66].

Leprosy was severely affected by the COVID-19 pandemic. Brazil has the second highest burden of leprosy in the world and has a high burden of COVID-19 [24]. There was a 41.4% reduction in the number of reported cases of leprosy between 2019 and 2020 as a direct result of cases not being identified, from closure of testing facilities and from fear of contracting COVID-19 keeping patients away from health care facilities [66, 67]. Delaying in treatment for leprosy has a direct impact on the severity of the disease and increase the likelihood of permanent disability [67, 68]. From a survey sent to forty-four leprosy centers, 16/20 or 80% responded that leprosy diagnostic services were reduced, only one responded that they had been closed; 7/8 (87%) of leprosy reconstructive surgery centers had suspended their services and active case finding 3/13 (23%) had been reduced, and 10/13 (77%) had been closed [69, 70]. In addition to the closing of active case finding and surgery centers, travel restrictions have had an impact on leprosy. Clinics in many places were sending patients home with 2 to 3 months of multi drug therapy (MDT) although reaction treatments of prednisolone or clofazimine have been unavailable or given out in regular one-month doses for those needing treatment [69, 70].

The impact on rabies programs around the world has been felt since the beginning of the pandemic. Based on a survey of 87 groups consisting of NGOs, government offices, and academics contributing from 47 countries, the threat is not only from the cancellation or postponement of dog vaccination programs [71]. Several countries have reduced their procurement of human rabies vaccines in 2020 and predict even lower procurement plans for 2021 and beyond [71]. If post exposure vaccines are not available, and bite centers are closed, people may go home and not seek further treatment as in a case in the Philippines where a patient went home untreated and died [71]. In 25% of responding countries staff who routinely conduct rabies surveillance were reassigned to COVID-19 response [72].

GAVI’s Vaccine Investment Strategy helps lower income countries obtain vaccines, in particular the human pre-exposure rabies vaccine; but this has been on hold due to COVID-19. In 2020 financial resources allocated to rabies were reduced by 60% and only 5% of reporting countries completed dog vaccination campaigns. Cities in Argentina, Cuba, Mexico, and Brazil all cancelled or postponed with no scheduled repeat day dog vaccination campaigns [73–76]. Haiti cancelled their dog vaccination campaign and the funds that were set aside for the campaign were diverted to COVID-19 [77]. In Arequipa Peru, due to cancellations, dog vaccine coverage was down to 12.3%, far lower than the 70% needed to stop transmission [78]. Models predicted that based on the decrease in dog vaccinations in Arequipa the rates of rabies cases would grow exponentially within months. December 2020 through March 2021 has tracked with the model predictions, with higher-than-average cases despite lower surveillance being performed [71]. Rabies is a disease that can be readily controlled by through dog vaccines, but the delays in the dog vaccination programs and the inability to obtain human rabies vaccines means that more people, often children under 15 years old, will be exposed and potentially die from rabies.

### Healthcare service disruption

While there is limited data on impacts of COVID-19 on progress towards control and elimination of the NTDs, day to day control of NTDs sits squarely at the healthcare interface and universally health care systems have been challenged by the COVID-19 pandemic. Many health systems in LMIC were already struggling pre-pandemic, with limited physical resources and a lack of physicians and nurses. The chronic and growing shortage of healthcare providers in LMIC due to increased work hours, low resources, and migration to HIC is not a new problem, but has been exacerbated by COVID-19, increasing workload, and causing burnout amongst doctors and nurses [79–82]. In Zimbabwe, even before the pandemic, there were strikes of medical professionals due to lack of protective equipment and basic tools such as gloves, bandages, and syringes [83, 84]. Doctors strikes and nursing shortages are affecting basic care as well as surveillance on NTDs. When there are a fraction of nurses performing the same tasks that were once done by a large team, underreporting of NTDs will be exacerbated with cases being undiagnosed [85].

Lockdown policies, albeit implemented in exceptional circumstances, kill people through disruption of health services and deprivation of livelihoods [86]. There have already been reported increases in maternal deaths during labor, as well as an increase in measles; essential healthcare being suspended or delayed and disrupting access to routine healthcare and/or preventative health care. With preventative campaigns postponed, cancelled, or shortened, the resulting increase in NTD cases and DALYs more generally have and will continue to derail progress towards elimination and meeting the targets set by the WHO 2030 Roadmap [86].

The WHO created a Pulse survey administered in 159 countries; in multiple configurations since July 2020 to assess the initial impacts of COVID-19 on healthcare systems [66, 87]. In addition to clinic closures and staff shortages respondents reported: disruptions due to essential medicines being out of stock (22% of 111 responding countries); unavailability of hospitals beds (19% of 111 responding countries); insufficient staff, often due to COVID-19 redeployment (66% of 112 responding countries; insufficient personal protective equipment in 26% of 111 countries [88].

More telling regarding the NTDs was the impact in health seeking behavior from within the community. Demand for services were lower than expected due to community fears and mistrust in seeking healthcare (57% of 112 responding countries), patients were observed to be not presenting for outpatient care (57% of 111 responding countries). There were perceptions that financial difficulties were affecting attendance (43% of 112 responding countries) in addition to access to care being prevented by travel restrictions (36% of 112 of responding countries) [88].

### COVID-19 and its consequences present an ongoing constraint to the management of NTDs

The lack of vaccine equity is ongoing despite the best efforts of WHO and partners. In 2022, 116 countries were still short of the target of 70% of the population vaccinated against COVID-19 [89] despite China having donated 1.3 billion of doses of their vaccines Sinovac and Sinopharm [20] and pledging 2 billion vaccine doses by the end of 2021 [90]. The pressure to vaccinate against COVID-19 has been intense, but this has drawn resource away from pre-pandemic vaccine programmes. By contrast COVAX, a WHO joint initiative with the Center for Epidemic Preparedness and Innovation (CEPI), Gavi- the Vaccine Alliance and UNICEF who are committed to facilitate donations of vaccines to countries in need but given vaccine shortages, brought about by the COVID-19 pandemic they were unable to reach their goal of 20% of the world’s population vaccinated by the end of the 2022 despite having successful distributed 1.99 billion vaccines to 146 countries so far, with more than 2 billion vaccines allocated for distribution by 2023 [91–94]. COVAX is unable to fulfill many of its 2021 pledges due to the purchasing of additional vaccines by HIC. The CEO of Pfizer, Albert Bourla, cited vaccine hesitancy in Africa as the reason for poor uptake of COVID-19 vaccination [95] despite communities in Africa being comfortable with childhood vaccination programmes that have eliminated smallpox and are very close to eliminating polio through vaccinations [96] and the reasons behind vaccine hesitancy remain debatable [97, 98]. Inequality persists with wealthy countries administering booster shots while poorer countries have insufficient vaccine to cover even 15% of their population [2, 99] and governments not wishing to accept short expiry vaccines [92, 100]. Ramping up of production of vaccines in the Global south remains an ambitious goal [101, 102]. The prioritization of profit over human health and lives has a direct impact on NTDs as the same pharmaceutical companies are the groups most capable of creating the next antibiotic, anti-malarial, anti-parasitic drugs needed to eliminate NTDs, particularly those that are becoming increasingly drug resistant.

### Poverty exacerbation due to COVID-19

It is estimated that as many as 97 million people in 2020 fell into poverty because of COVID-19. While lower than the predicted 119 million this is a “historically unprecedented increase in global poverty” [103, 104] derailing progress towards the SDGs and undoing 5 years of progress towards poverty elimination with LMIC hit harder. With an increase in poverty comes an increase in the diseases of poverty.

The NTDs are diseases of poverty, affecting poor rural communities that are often dependent on tourism, the neglected zoonotic diseases affect communities dependent on wildlife and animal trade for their livelihoods. In sub-Saharan Africa (SSA), Kenya, Nigeria, Uganda, and Ghana are in the top ten countries in Africa that account for the most tourism dollars. In SSA T&T contribution to the GDP declined by 46.5% between 2019 and 2020, resulting in a USD 48.8 billion loss to the GDP and 5.7 million jobs lost [105]. People living in poverty have been required to make hard choices between taking unnecessary health risks and providing for their families [106].

Lockdowns have had an unprecedented negative impact on children of school age particularly in LMIC. While school closures were global, although in HIC many schools and children were able to continue education online via online platforms, an estimated 1.5 billion children were left without access to education [107]. Globally, only one in three children has access to internet at home, making online education unattainable [108]. It is anticipated that as many as 5 million children will not be able to return to school due to teen pregnancy, lack of school fees, or needing to help the family by working [109, 110].

Uganda and the Philippines had two of the longest school shutdowns due to COVID-19 and since many MDA programs are delivered to children in schools, this creates a delay in the treatment and elimination of diseases [108]. In Uganda schools only reopened in January of 2022 after two years of closures; 15.5 million Ugandan students lost two years of education as many lack the resources to participate in online schooling. These children will also lose out on any future MDA programs that will be restarted by the schools, contributing to millions of missed opportunities to end transmission of NTDs. In addition to the missed educational opportunities which impact the children’s future ability to move ahead in life, as many as 370 million children lost their access to the only reliable meal of the day which was provided by the schools [107].

## Conclusions

The COVID-19 pandemic profoundly impacted global health research and global health equity. Countries affected by one or more Neglected Tropical Disease were as expected, have been affected by economic crises, lockdowns, vaccine inequities, and disruption to health services supply shortages all which impact on their ability to manage the NTDs and follow pathways to elimination and control. Attention and funding were diverted from all sectors, significantly affecting research and development efforts set out in the World Health Organization's NTD elimination Roadmaps. The direct impacts of COVID-19 can be seen from the restructuring of investments in research and development for the NTDs.

As is often the case, the bottom billion are the most affected the most by the global turmoil brought on by the COVID-19 pandemic and subsequent economic crises. Ongoing challenges in funding for the NTDs, for research and development will inevitably push back targets and impede development of new tools to manage these diseases. Economic crises, logistics and supply chain disruptions as well as deepening poverty have put a strain on already weak healthcare systems and exacerbated delivery of programmes aimed at alleviating the suffering caused by the NTDs. In particular, the delays and constraints posed to NTD management and elimination programs will have long-reaching consequences. The shortage in medical staff, doctors, nurses, and community health care workers will continue to impact LMICs’ ability to care for the most neglected and underserved portions of the global population.

Not only does an increase in global poverty set back progress in our ability to tackle the NTD’s but it also creates a situation where more people are at risk for developing NTDs; with vector control programmes and MDA suspended.

COVID-19 has shed light on issues with infrastructure, and the need for robust health care systems to be ready for the next pandemic. It has also emphasized the importance of global action for health. COVID-19 will remain an issue for so long as countries are unable to vaccinate their populace and provide adequate consistent healthcare. Poverty and NTDs are intertwined in so many ways that eliminating one is extremely unlikely without eliminating the other.

### Supplementary Information

Supplementary Material 1.

Supplementary Material 2.

## Data Availability

The datasets generated and/or analysed during the current study are available from the corresponding author on reasonable request.

## Abbreviations

ASCEND: Accelerating the Sustainable Control and Elimination of Neglected Tropical Diseases
Big 3: HIV/AIDS, malaria and tuberculosis
CEPI: Center for Epidemic Preparedness and Innovation
FCDO: Foreign Commonwealth and Development Office
FDA: Federal Drug Administration
GDP: Gross Domestic Product
HIC: High Income Countries
HIV/AIDS: Human Immunodeficiency Virus/Acquired Immune Deficiency Syndrome
LMIC: Low- Middle- Income Countries
MDA: Mass Drug Administration
MDT: Multi Drug Therapy
MRC: Medical Research Council
NIH: National Institutes of Health
NTD: Neglected Tropical Disease
PC Tracker: Policy Cures Research and Development Tracker
R&D: Research and Development
SDG: Sustainable Development Goals
SSA: Sub Saharan Africa
T & T: Tourism and travel
WASH: Water, Sanitation and Hygiene
WHO: World Health Organization
