# Supplementary material for: Impact of COVID-19 on the neglected tropical diseases: a scoping review
Source: Infect Dis Poverty. 2024 Jul 29;13:55. doi: 10.1186/s40249-024-01223-2 (PMC11285209; doi:10.1186/s40249-024-01223-2)
Supplement: Supplementary file 1 — Supplementary Material 1. [file 40249_2024_1223_MOESM1_ESM.docx]

Appendix: Impact of COVID-19 on the Neglected Tropical Diseases: A scoping review – Protocol

# Authors

Caitlin Brigid Butala ^1,2^, Roo Nicola Rose Cave ^1^, Jenna Fyfe ^1^, Paul Gerard Coleman ^1^, Guo-Jing Yang ^3^, Susan Christina Welburn ^1,2,3,4,*^

^1^Infection Medicine; Edinburgh Medical School: Biomedical Sciences, College of Medicine and Veterinary Medicine, The University of Edinburgh, 1 George Square, Edinburgh EH8 9TB, Scotland, United Kingdom.

^2^Zhejiang University – University of Edinburgh Institute: Biomedicine, Zhejiang University School of Medicine, 718 East Haizhou Road, Haining, 314400, People’s Republic of China.

^3^Key Laboratory of Tropical Translational Medicine of Ministry of Education, The School of Tropical Medicine, The First Affiliated Hospital, Hainan Medical University, Haikou 571199, Hainan, People’s Republic of China.

^4^School of Global Health, Chinese Centre for Global Tropical Disease Research, Shanghai Jiao Tong University School of Medicine, Shanghai 200025, People’s Republic of China.

^*^ Corresponding author: Susan Christina Welburn; email: [sue.welburn@ed.ac.uk](mailto:sue.welburn@ed.ac.uk)

# Abstract

**Objective:** This study aims to investigate the impact of the COVID-19 pandemic on the prevalence, management, and control of neglected tropical diseases (NTDs) highlighting the current or prospective impact of COVID-19 on NTD programmes.

**Introduction:** These 21 diseases can cause lifelong disabilities and impairments but historically garner less attention and funding than the Big 3; Tuberculosis, Malaria, and Human Immunodeficiency Virus/Acquired Immune Deficiency Syndrome (HIV/AIDS).

**Inclusion criteria:** Inclusion criteria will be health policy makers, health programs, ministries of health, NGOs, philanthropists, Official Development Aid donor countries that are working in connection with NTDs; health policies, programmes, interventions, diagnostics, treatments, and management focused on NTDs; work contributing to the management, monitoring, or elimination of NTDs where the impact of COVID-19, negative, positive or neutral must be discussed.

Exclusion criteria will be health policies, health programmes, interventions, diagnostics, treatments, management not addressing diseases listed as NTDs; discussion of work on NTDs with no discussion of the impact of NTDs.

**Methods:** Using open-source available data from policy and documentation from official websites of the relevant stakeholders, we explored the challenges posed by the pandemic in maintaining existing Neglected Tropical Diseases (NTD) control programs; the disruptions to healthcare services, reduction of finance and the potential long-term implications and consequences for those poorer, neglected populations in Low- Middle-Income-Countries (LMICs), disproportionately affected by the NTDs. We identified specific NTDs affected by the COVID-19 pandemic, and examined disruptions caused to on ongoing NTD control and elimination programs. We further undertook an evaluation of the socio-economic factors exacerbating the impact of COVID-19 on NTD burden.

**Results:** this is the scoping review protocol; results are found in “Impact of COVID-19 on the Neglected Tropical Diseases: A scoping review”

**Conclusions:** this is the scoping review protocol; conclusions are found in “Impact of COVID-19 on the Neglected Tropical Diseases: A scoping review

**Keywords:** Neglected Tropical Diseases, Funding, WHO, Research and Development, Investment, DALYs, Covid-19.

# Introduction

This study investigates the impact of the COVID-19 pandemic on the prevalence, management, and control of neglected tropical diseases (NTDs) highlighting the current or prospective impact of COVID-19 on NTD programmes. These 21 diseases can cause lifelong disabilities and impairments but historically garner less attention and funding than the Big 3; Tuberculosis, Malaria, and Human Immunodeficiency Virus/Acquired Immune Deficiency Syndrome (HIV/AIDS).

COVID-19 has had a crippling effect on NTDs from several fronts and angles. Like many aspects of eliminating NTDs, this is a multi-dimensional problem that requires a complex solution. Understanding the impact of COVID-19 on NTDs and their programmes is critical to progression towards elimination.

# Scoping review question

This scoping review explores changes to funding distribution, economic circumstances, logistics and supply chain disruptions in the context of limited Low-Middle Income Country (LMIC) healthcare systems. This aims to inform a commentary on the impact COVID-19 has had on the research and development efforts set out in the World Health Organization's NTD elimination Roadmaps.

# Inclusion criteria

| **SPIDER/PICO** | SPIDER: sample, phenomenon of interest design, evaluation, research type  PICO: patient/population, intervention, comparison, and outcomes. |
| --- | --- |
| Population/Sample | Health policy makers, health programs, ministries of health, NGOs, philanthropists, Official development aid donor countries, that are working in connection to NTDs or the big 3 (HIV, TB, Malaria). |
| Intervention/Phenomenon of Interest | NTD focused: health policies, health programmes, interventions, diagnostics, treatments, management |
| Comparison/Design | Any |
| Outcome/Evaluation | The impact of Covid-19, negative, positive, or neutral must be discussed. |
| Study Type/Research Type | Any |

| **SPIDER** | **Inclusion Criteria** | **Exclusion Criteria** |
| --- | --- | --- |
| Population/Sample | Health policy makers, health programs, ministries of health, NGOs, philanthropists, Official development aid donor countries, that are working in connection with NTDs. | Organisations and programmes working on other healthcare or non NTD diseases. |
| Intervention/  Phenomenon of Interest | Health policies, health programmes, interventions, diagnostics, treatments, management focused on NTDs | Health policies, health programmes, interventions, diagnostics, treatments, management not addressing diseases not listed as NTDs. |
| Comparison/Design | Include all | None |
| Outcome/Evaluation | Work contributing to management, monitoring, or elimination of NTDs where the impact of COVID-19, negative, positive, or neutral must be discussed. | Discussion of work on NTDs with no discussion of the impact of COVID-19 |
| Study Type/Research Type | Include all | None |
| Other/Extras | Health policy makers, health programs, ministries of health, NGOs, philanthropists, Official development aid donor countries, that are working on funding in connection to the big 3 (HIV, TB, Malaria).  Funding relating to NTDs and the big 3. | Publications whose focus is on grey work and women in tourism, these papers sometimes reference the impact of NTDs and additional challenges due to COVID-19, but unless the focus is on NTDs and COVID-19 the publication should be excluded. |

# Methods

Scoping review was conducted in full accordance with the JBI methodology for scoping reviews.

### Search strategy

Databases:

- PubMed
- Web of Science
- Jstor
- Science Direct
- Google Scholar

All publications extracted deduplicated and uploaded to Mendeley.

Hand Searching:

- Bill and Melinda Gates Foundation
- Devex
- DnDi
- FDA
- GAVI
- Global Citizen
- Global Fund
- Google and Google Scholar
- Johns Hopkins
- News providers: Reuters, CNN, BBC, Forbes, NPR, The Guardian
- NIH
- Our world in data
- Policy Cures G-Finder Report
- Relief Web
- Save the Children
- Sci
- Stop TB partnership
- Wellcome Trust
- WHO, PAHO
- World Bank
- Uniting to combat NTDs
- UN
- Unicef

All publications extracted deduplicated and uploaded to Mendeley.

Search Terms

Searches were constructed by combining search terms from Table 1. For the NTD and Big 3 searches respectively one or more search term was used from each word group. Words within a word group were combined with OR. AND was used between word groups.

| **Search word groups** | **NTD Search** | **Big 3 Search** |
| --- | --- | --- |
| **1** | Lymphatic filariasis, leishmaniasis, echinococcus, yaws, dengue, soil transmitted helminths (ascariasis, trichiuriasis, hookworm, whip worm, roundworm, strongyloidiasis), mycetoma, Buruli ulcer, guinea worm, rabies, scabies, trachoma, human African trypanosomiasis (HAT, sleeping sickness), chagas, leprosy, schistosomiasis, onchocerciasis, snakebite envenoming, foodborne trematodiases, taeniasis, cysticercosis, noma | HIV, AIDS, Tuberculosis, TB, Malaria, HIV/AIDS |
| **2** | COVID-19, COVID | COVID-19, COVID |
| **3** | Economic, economics, economic evaluation, funding, financing, delay, budget cuts, lockdowns, vaccines, tourism | Economic, economics, economic evaluation, funding, financing, delay, budget cuts, lockdowns, vaccines, tourism |

Table 1: Search terms for inclusion and exclusion to scoping review.

### Study/Source of evidence selection

Database search method:

1. Searches performed in December 2020 (with an update performed in June 2021, September 2022, December 2023 and April 2023)* in databases specified in above (Search Strategy).
2. Publications extracted and uploaded to Mendeley.
3. Publications extracted from Mendeley to be uploaded to a deduplicator software (https://sr-accelerator.com/#/deduplicator).
4. Publications deduplicated.
5. Publications extracted and re-uploaded to Mendeley.
6. Publications ready for abstract screening by CB and team.

* Initial database searches were performed in December 2020 with searched updated over the specified time with a final updated search performed in December 2023 and additional search added in April 2023 to account for addition of Noma to NTD list by WHO in December 2023.

Reference/Snowball search method:

Snowball searching of citations, was undertaken in which all articles that were included had their references searched for articles that would fit our inclusion criterion. The reference list from each publication extracted from the databases was read, analysed and publications of note (not already extracted as part of the database search) were uploaded to Mendeley for screening.

Hand search method:

Open-source available data from policy and documentation were searched on websites of the relevant stakeholders including NGOs, LMIC governments, philanthropic groups. Information was searched through government foreign aid policies, grant proposal and awards, NGO annual report statements, and news articles found through Google Search. When selecting searching here, the following key words were used, projects mentioning WASH, NTD, Neglected Tropical Diseases, or any specific disease name on the WHO NTD list in relation to COVID-19 or budget cuts. All sources were uploaded to Mendeley for screening.

### Project Tools

- Microsoft Word
- Microsoft Excel
- Microsoft PowerPoint
- Mendeley
- Deduplication software (https://sr-accelerator.com/#/deduplicator)
- R studio

### Data extraction

Method of collection

Each included publication had its key findings recorded in Microsoft Excel:

- Financial data
- Key discussion points
- Opinion of author/healthcare provider/public health body/political representative/NGO etc.
- Key dates
- Information on research and development funding distribution, health or country/regional economics, logistics and supply chain information
- Healthcare or NTD programme provision
- Other as is found through screening.

Extraction conducted by: Caitlin Butala.

### Data analysis and presentation

The key findings from included studies collected in Microsoft Excel by CB is described above in the Data Extraction section. Information will form the basis of discussion topics and data collated for figures. This information will be grouped into the following discussion topics:

- COVID-19 Research Funding.
- Research and development funding for NTDs and any rerouting of this funding to COVID-19 research.
- Foreign aid and health budgets and cuts in the UK.
- The impact of COVID-19 on other diseases e.g. TB and Malaria, and how this will impact disease research and development in general as well as how it will impact NTDs.
- Discussion around lockdowns and closures of healthcare services.
- The impact of lockdown on healthcare accessibility especially in LMICs.
- Vaccination impacts, including COVID-19 vaccination.
- Poverty affecting access to healthcare and the impact of COVID-19.

This information was collated to create the following figures:

- Research and Development funding allocated to NTDs (see list above), HIV/AIDS, Malaria and Tuberculosis, pre-2019 and up to 2022.
- Research and Development funding allocated to individual NTDs pre-2019 and up to 2022.
- Information on UK government budgets including any changes and cuts (health funding and foreign aid funding in particular).

# Acknowledgements

The authors alone are responsible for the views expressed in this article and they do not necessarily represent the views, decisions, or policies of the institutions with which they are affiliated.

# Funding

This research was supported by the National Institute for Health Research (NIHR) Global Health Research programme (16/136/33) using UK aid from the UK Government. The views expressed in this publication are those of the author(s)and not necessarily those of the NIHR or the Department of Health and Social Care (SCW, CBB). This work was supported by the Zhejiang University Education Foundation Emergency Research Fund (SCW), Zhejiang University and the University of Edinburgh (CBB).

# Declarations

The datasets generated and/or analysed during the current study are freely available from the corresponding author on reasonable request.

# Author contributions

CBB, SCW and PGC conceived this study. SCW, PGC, JF and GY supervised the study. CBB conducted data collection, data curation, and formal analysis and wrote the first draft. NRC undertook data analysis. All authors read and approved the final manuscript.

# Conflicts of interest

None

# References

TBD when publication is submitted.

Protocol template provided by JBI Scoping Review Network at <https://jbi.global/scoping-review-network/resources>.
