# Supplementary figures and images for: Impact of COVID-19 on the neglected tropical diseases: a scoping review
Source: Infect Dis Poverty. 2024 Jul 29;13:55. doi: 10.1186/s40249-024-01223-2 (PMC11285209; doi:10.1186/s40249-024-01223-2)

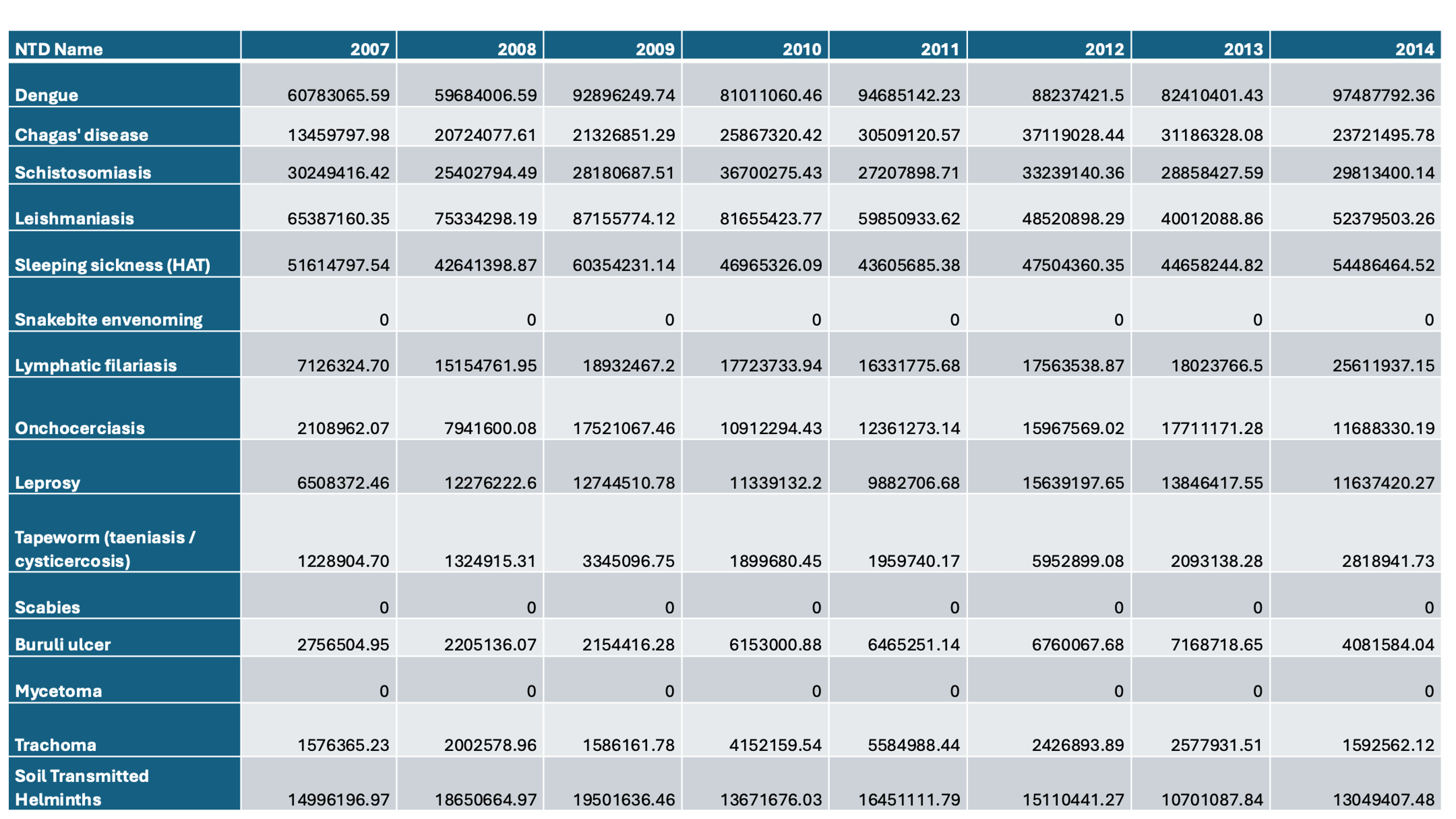


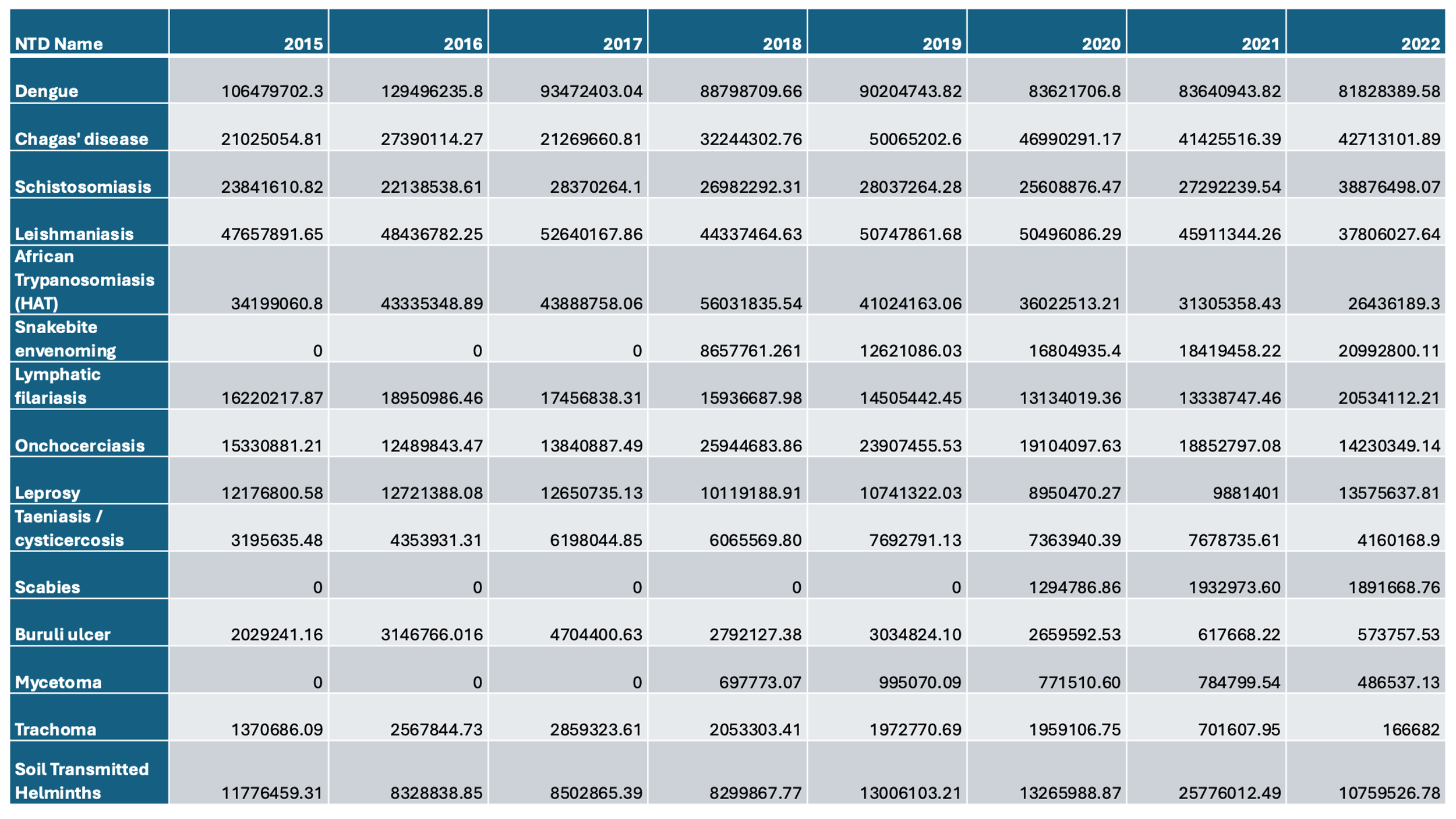

Supplement: Supplementary file 2 — Supplementary Material 2. [file 40249_2024_1223_MOESM2_ESM.docx]
